# The Free-Living Stage Growth Conditions of the Endophytic Fungus *Serendipita indica* May Regulate Its Potential as Plant Growth Promoting Microbe

**DOI:** 10.3389/fmicb.2020.562238

**Published:** 2020-09-22

**Authors:** Teresa Dias, Vívian Pimentel, Antônio Jesus Dorighetto Cogo, Raquel Costa, Amanda Azevedo Bertolazi, Camila Miranda, Sávio Bastos de Souza, Juliana Melo, Manuela Carolino, Ajit Varma, Frederico Eutrópio, Fábio Lopes Olivares, Alessandro Coutinho Ramos, Cristina Cruz

**Affiliations:** ^1^Centre for Ecology, Evolution and Environmental Changes, Faculdade de Ciências, Universidade de Lisboa, Lisbon, Portugal; ^2^Laboratory of Physiology and Biochemistry of Microorganisms, Universidade Estadual do Norte Fluminense, Campos dos Goytacazes, Brazil; ^3^Laboratory of Environmental Microbiology and Biotechnology, Universidade Vila Velha, Vila Velha, Brazil; ^4^Plant Physiology Lab, Universidade Estadual do Norte Fluminense, Campos dos Goytacazes, Brazil; ^5^Amity Institute of Microbial Technology, Amity University Uttar Pradesh, Noida, India; ^6^Faesa Centro Universitário – Campus Vitória, Vitória, Brazil; ^7^Cell Tissue and Biology Lab, Universidade Estadual do Norte Fluminense, Campos dos Goytacazes, Brazil

**Keywords:** free-living stage, fungal phenotype, morphology, physiology, plant-growth-promoting-microbes, symbiosis stage

## Abstract

*Serendipita indica* (former *Piriformospora indica*) is a non-obligate endophytic fungus and generally a plant growth and defence promoter with high potential to be used in agriculture. However, *S. indica* may switch from biotrophy to saprotrophy losing its plant growth promoting traits. Our aim was to understand if the free-living stage growth conditions (namely C availability) regulate *S. indica*’s phenotype, and its potential as plant-growth-promoting-microbe (PGPM). We grew *S. indica* in its free-living stage under increasing C availabilities (2–20 g L^–1^ of glucose or sucrose). We first characterised the effect of C availability during free-living stage growth on fungal phenotype: colonies growth and physiology (plasma membrane proton pumps, stable isotopic signatures, and potential extracellular decomposing enzymes). The effect of the C availability during the free-living stage of the PGPM was evaluated on wheat. We observed that C availability during the free-living stage regulated *S. indica*’s growth, ultrastructure and physiology, resulting in two distinct colony phenotypes: compact and explorer. The compact phenotype developed at low C, used peptone as the major C and N source, and displayed higher decomposing potential for C providing substrates; while the explorer phenotype developed at high C, used glucose and sucrose as major C sources and casein and yeast extract as major N sources, and displayed higher decomposing potential for N and P providing substrates. The C availability, or the C/N ratio, during the free-living stage left a legacy to the symbiosis stage, regulating *S. indica*’s potential to promote plant growth: wheat growth promotion by the explorer phenotype was ± 40% higher than that by the compact phenotype. Our study highlights the importance of considering microbial ecology in designing PGPM/biofertilizers. Further studies are needed to test the phenotypes under more extreme conditions, and to understand if the *in vitro* acquired characteristics persist under field conditions.

## Introduction

To maintain the current human population growth and consumption patterns, food demand is forecasted to double by 2050 (Rockström et al., 2009), while its environmental footprint must be reduced (for the European Union, see Directive 2009/128/EC regarding the sustainable use of pesticides). This creates the need for cleaner farming practices capable of boosting crop yields while decreasing environmental impacts (Dias et al., 2015, 2018).

So far, conventional farming has been relying mostly on a large use of chemical fertilisers and pesticides, which represents significant production costs, and poses health and environmental risks and damages (Le Cocq et al., 2017; Jiang et al., 2020). As the appreciation of the vital role of soil life in farming sustainability is increasing (Bender et al., 2016; Le Cocq et al., 2017; Murphy et al., 2018), so is the use of biological approaches (e.g., biofertilisers; products containing soil microbes to promote plant growth – Herrmann and Lesueur, 2013). Biofertilizers based on arbuscular mycorrhizal fungi (AMF) are of special interest because most major crops (e.g., maize, wheat, and soybean) form associations with AMF, which are a permanent and natural component of agrosystems (Dias et al., 2018). Besides the well-known improvement in plant nutrition (Dias et al., 2015), AMF also promote pathogen suppression, pollination, herbivore protection, and improved water relations (Verbruggen and Kiers, 2010). However, AMF are obligate biotrophs, which means that AMF require the plant host to grow and to complete their life cycle (Smith and Read, 2008). Consequently, the propagation step must include a phase of cultivation with the plant host which is costly, time and space-demanding (Berruti et al., 2016). Alternatively, non-obligate biotroph fungi that confer benefits to the plant host similar to those conferred by AMF, are especially attractive as plant-growth-promoting-microbes (PGPM) and potential biofertilizers.

The endophytic fungus *Serendipita indica* (former *Piriformospora indica*) fulfils the abovementioned advantages over AMF, and displays a high versatility of association with plants (Varma et al., 1999; Bertolazi et al., 2019; Heidarianpour et al., 2020; Leyva-Rojas et al., 2020). Further, *S. indica* grows inter- and intracellularly and forms a spiral structure similar to AMF’s arbuscules, creating a symbiotic interface in the space between fungal hyphae and the cell wall (Schäfer and Kogel, 2009). As a result, *S. indica* is a model for studying symbiosis (Varma et al., 2012).

The absorption and bidirectional exchange of nutrients between symbiont partners occurs through specific transporters at the symbiotic interface in both AMF (Wang et al., 2014; Lanfranco et al., 2018) and *S. indica* (Zuccaro et al., 2014; Rani et al., 2016): inorganic phosphate and carbohydrates are the main nutrients exchanged at these interfaces, where the fungus supplies the necessary phosphate to the plant host, which in turn provides carbon (C – hexoses) to the fungus. Fungal cells use glucose as the preferred C source (Boldt et al., 2011). Both high C (sucrose) and high phosphate availabilities negatively impact fungal and symbioses development in AMF (Mugnier and Mosse, 1987). Previous studies have identified 15 putative genes of hexose transporters in the genome of the ectomycorrhizal fungus *Laccaria bicolor* (Lopez-Pedrosa et al., 2006), and high affinity hexose transporters in other fungi such as *Amanita muscaria* (AmMST1 – Smith et al., 2001), *Tuber borchii vittadini* (TbHXT1 – Polidori et al., 2007), and *S. indica* (PiHXT5 – Rani et al., 2016). The *S. indica*’s high affinity hexose transporter PiHXT5 is regulated during symbiosis and responds distinctly to different glucose availabilities (Rani et al., 2016), which suggests that C availability may be an important factor for hyphae development and the expression of functional traits in *S. indica*. Many studies report *S. indica’s* ability to promote plant growth (Varma et al., 1999, 2012; Singh et al., 2000; Waller et al., 2005; Qiang et al., 2012; Li et al., 2017; Xu et al., 2018). However, the above-mentioned observations raise important questions: (i) do free-living stage growth conditions (namely C availability) regulate *S. indica*’s phenotype? And (ii) can the free-living stage growth conditions regulate *S. indica*’s potential as PGPM during the symbiosis stage? Answering these questions could help explain and understand why *S. indica* may display different behaviours (switching from biotrophic to saprophytic – Lahrmann et al., 2013), and why inoculation with *S. indica* does not always provide benefits to the plant host (Kaldorf et al., 2005; Zuccaro et al., 2011).

To answer these questions, we grew *S. indica* in its free-living stage (*in vitro*) under increasing C availabilities (2, 5, 10, 15, and 20 g L^–1^ of glucose or sucrose). We hypothesised that increasing C availability during the free-living stage growth would trigger nutrient imbalances, resulting in fungal physiological changes (i.e., more adapted to acquire the limiting nutrients), that later would regulate its plant growth promoting potential in the symbiosis stage.

We first characterised the effect of increasing C availability during *S. indica*’s free-living stage (*in vitro* growth) on its phenotype: colonies growth (biomass, spores size, and ultrastructure) and physiology (C and N stable isotopic signatures and potential extracellular enzyme activities related with the decomposing potential of carbon, nitrogen, and phosphorus). Increased activity and expression of plasma membrane proton pumps (P-H^+^-ATPase hydrolyses ATP to transport H^+^ from the cytosol to the extracellular medium – Palmgren and Nissen, 2011) is associated with the bi-directional exchange of nutrients (including hexoses) in fungal and plant cells (Palmgren, 2001). Glucose plays an important role in the modulation and expression of P-H^+^-ATPase in yeast (Eraso and Gancedo, 1987; Portillo, 2000) and in filamentous fungi, including basidiomycetes (Ramos et al., 2005; Kralj Kuncic et al., 2013) but little is known on the effect of glucose on fungi capable of associating with plants. Therefore, the effect of C availability on *S. indica* P-H^+^-ATPase was one of the physiological aspects we studied. Finally, we evaluated the effect of the C availability (glucose) during the free-living stage (*in vitro* growth) on its plant promoting potential during the symbiosis stage by analysing wheat’s growth (biomass) and root’s fungal colonisation and potential extracellular enzyme activities related with the decomposing potential of carbon, nitrogen, and phosphorus.

## Materials and Methods

### Effect of the C Availability During the Free-Living Stage on *S. indica*’s Phenotype

We tested the effect of increasing C availabilities (2, 5, 10, 15, and 20 g L^–1^) on the growth and activity of *S. indica* cultures. We tested two widely used C sources: glucose and sucrose. Combining the two C sources and the five C availabilities we tested 10 combinations. Each treatment was replicated three times.

#### *S. indica*’s *in vitro* Cultivation

The endophytic fungus *S. indica* was obtained from the culture collection of the Centre for Ecology, Evolution and Environmental Changes, at Faculdade de Ciências, Universidade de Lisboa (Portugal). The stock cultures were propagated in solid potato dextrose agar (PDA), pH 6.5 at 28°C for 15 days. Since *S. indica* produces pear-shaped chlamydospores, which stay attached to the mycelium, spores were removed by adding 100 μL of Tween 80–10 mL of the PDA culture broth containing the mycelium. Each sample was ground with an Omni-Mixer Homogenizer (Solvall^®^, Norwalk, CT, United States) during five cycles of 3 min and sonicated (Elmasonic S30, Elma^®^, Germany) for 5 min, as described by Kumar et al. (2011). Mycelium was then separated from the culture medium, washed with sterile distilled water and dried using filter paper. Mycelia were homogenised in 40 mL of sterile NaCl solution 0.85% (w/v), and the homogenate analysed under the microscope to determine spore concentration.

Approximately 20 *S. indica* spores were inoculated into 100 mL Erlenmeyer flasks containing 25 mL of liquid modified Hill–Kaefer (KM) broth medium pH 6.5 (Hill and Kaefer, 2001), which contains peptone (2 g L^–1^), casein (1 g L^–1^), yeast extract (1 g L^–1^), and glucose or sucrose (2, 5, 10, 15, and 20 g L^–1^) as C sources. Media were autoclaved for 20 min at 121°C and 1 atm. *S. indica* cultures were grown at 28°C and 125 rpm for 11 (morphological and physiological studies) or 2 days (electrophysiological studies), with three biological replicates per C source (glucose or sucrose) and concentration.

#### *S. indica*’s Growth Characterisation

For biomass determination (and isotopic signatures), each 11-day old *S. indica* culture was harvested, washed with sterile distilled water and lyophilized (Model Alpha1-5, Christ^®^). For colony characterisation, 11-day old *S. indica* cultures were harvested, washed with sterile distilled water and each colony was visually characterised for its size, texture, colour, shape, and surface type. This was done for two contrasting C availabilities with both C sources: 2 and 20 g L^–1^ of glucose or sucrose. We determined spores’ size by staining the spores with lactophenol cotton blue 0.5% (w/v) and measuring its size using a micrometre ocular at the optical microscope (Olympus BX51TF, Japan). Thirty spores per replicate (90 spores per treatment) were measured. Spores were classified as small (maximum length 15 μm) or large (maximum length > 15 μm).

#### *S. indica*’s Ultrastructure

Samples from *S. indica* mycelium were fixed in an aqueous solution containing 2.5% glutaraldehyde and 4.0% formaldehyde diluted in 0.05 M sodium phosphate buffer pH 7.2, at room temperature for 2 h. Subsequently, they were washed three times for 20 min each, in 0.05 M phosphate buffer pH 7.2 and subjected to an increasing ethanolic concentration series (30, 50, 70, 90, 100, 100% v/v) for 30 min in each step. Samples were then infiltrated with LR White methacrylate resin medium grade (London Resin Company, United Kingdom), mounted in gelatin capsules filled with fresh resin and polymerised overnight in an oven at 60°C under anaerobic catalysis. Cured capsules containing the samples were cut with an ultramicrotome (Ultracut E II Reichert-Jung). Ultra-thin sections (50–70 nm) were obtained with a diamond knife, collected with a formvar-coated copper grid (300 mesh), and stained with uranyl acetate and lead citrate for TEM studies. The grids were viewed and photographed using a transmission electron microscope (Philips CM 100, Royal Philips Electronics, Amsterdam).

#### *S. indica*’s Membrane Isolation and ATP-Dependent H^+^ Transport

Total membrane isolation was adapted from the methodology described by Okorokov and Lehle (1998). Fungal mycelium was separated from the liquid KM medium using sterile gaze and transferred to a glass potter containing 20 mL of ice-cold buffer (12.5% sucrose, 20 mM 3-(N-morpholino)propanesulfonic acid (MOPS-KOH) pH 7.6, 1 mM DTT, 1 mM benzamidine, 1 mM PMSF, cocktail of protease inhibitors and 0.3% BSA). After 21 complete cycles of homogenization, the mixture was centrifuged for 5 min at 4°C (20,000 *g*). The supernatant was collected and centrifuged at 100,000 *g* for 45 min at 4°C. The pellet was resuspended in 1.5 mL of ice-cold buffer (12.5% sucrose, 20 mM MOPS-KOH pH 7.6, 1 mM DTT, 1 mM benzamidine and cocktail of protease inhibitors), aliquots were taken and stored at −70°C.

ATP-dependent H^+^ transport was measured in membrane vesicles isolated from the *S. indica* mycelium according to Okorokov and Lichko (1983). H^+^ gradient was monitored as the initial rate of fluorescence quenching of 9-amino-6-chloro-2-methoxyacridine (ACMA) at 25°C in a fluorimeter (model F-3010, Hitachi, Tokyo). The excitation wavelength was set at 415 nm and the emission wavelength was set at 485 nm. Total membrane suspension (50 μg) was transferred to the incubation buffer containing 12.5% sucrose, 20 mM MOPS-KOH pH 6.5, 1 mM ACMA, 2.5 mM MgSO_4_ and 50 mM KCl. The reaction was initiated by the addition of 1 mM ATP pH 6.5. The addition of 20 mM NH_4_Cl was used to show a recovery of the fluorescence that indicated a collapse of the preliminarily formed H^+^ gradient. The P-H^+^-ATPase proton pumping activity was measured with and without vanadate (0.2 mM Na_3_VO_4_, a reversible inhibitor – Wang et al., 2013) and the orthovanadate-sensitive activity was attributed to the P-H^+^-ATPase. Maximum fluorescence (F_max_) reflects the steady-state amplitude of the ΔH formation and it was calculated as ΔF/F and expressed as percentage. Initial velocity of H^+^ transport (*V*_0_) was calculated by an extrapolation of the fluorescence quenching curve for 1 min.

#### *S. indica*’s Nitrogen Concentration and Stable Isotopic Signatures

*Serendipita indica*’s mycelia grown under increasing C availabilities were analysed for C and nitrogen (N) stable isotope ratios at the Stable Isotopes and Instrumental Analysis Facility, Universidade de Lisboa (Portugal), using approximately 1 mg of lyophilized ground (Retsch MM 2000, Germany) material. Sample ^13^C/^12^C and ^15^N/^14^N ratios were determined by continuous flow isotope mass spectrometry (Preston and Owens, 1983), on a Hydra 20–22 (Sercon, United Kingdom) stable isotope ratio mass spectrometer, coupled to an EuroEA (EuroVector, Italy) elemental analyser for online sample preparation by Dumas combustion. The standards used were IAEA-N1 and USGS-35 for N isotope ratio, and IAEA-CH6 and IAEA-CH7 for C isotope ratio; δ^15^N results were referred to Air and δ^13^C to PeeDee Belemnite (PDB). Precision of the isotope ratio analysis, calculated using values from six to nine replicates of laboratory standard material interspersed among samples in every batch analysis, was ≤ 0.2‰. We also analysed individually the δ^13^C and δ^15^N of the C (peptone, yeast extract, casein and glucose or sucrose) and N (peptone, casein and yeast extract) sources present in the growth media. Mycelium N and C concentrations (w/w) were determined on the same samples as the isotopic signatures using the major mass signal, with L-Cystine OAS and Methionine OAS certified elemental reference materials (Elemental Microanalysis, United Kingdom) as calibration standards.

#### *S. indica*’s Potential Extracellular Enzyme Activities

*Serendipita indica*’s mycelia grown under increasing C availabilities were analysed for their potential extra cellular enzyme activities related with the decomposing potential of C (β-glucosidase, β-glucuronidase, β-xylosidase, and cellobiohydrolase), N (N-acetylglucosaminidase, and Leucine aminopeptidase), and phosphorus (acid phosphatase). Decomposing potentials of C and N corresponded to the sum of the respective enzyme activities.

All enzyme assays were performed in 96-well filter plates (AcroPrep^TM^ 96-filter plate with 30–40 μm mesh size; Pall Life Sciences, Crailsheim, Germany) as described by Pritsch et al. (2011). Briefly, both the samples and the blanks were incubated in 150 μL incubation buffer on a microplate shaker at room temperature. At the end of incubation time of each enzyme assay, incubation solutions were transferred to measurement plates (black microplates, Nunc, Langenselbold, Germany) using a vacuum manifold and 100 μL of stopping buffer (pH 11) was added. In between enzyme assays, samples where rinsed with 150 μL of rinsing buffer. Fluorescence was measured at 360 nm excitation and 460 nm emission using the fluorescence microplate reader (BioTek^TM^ FLx800^TM^). After the seventh enzyme assay, *S. indica*’s mycelium was washed with sterile distilled water, and dried at 50°C until constant weight. Enzyme activities were calculated based on the emitted fluorescence and were expressed as μmol h^–1^ of released substrate per gramme of mycelium (Pritsch et al., 2011).

### Effect of *S. indica*’s Free-Living Stage Growth Conditions on Its Potential as PGPM During the Symbiosis Stage

**FIGURE 1:**
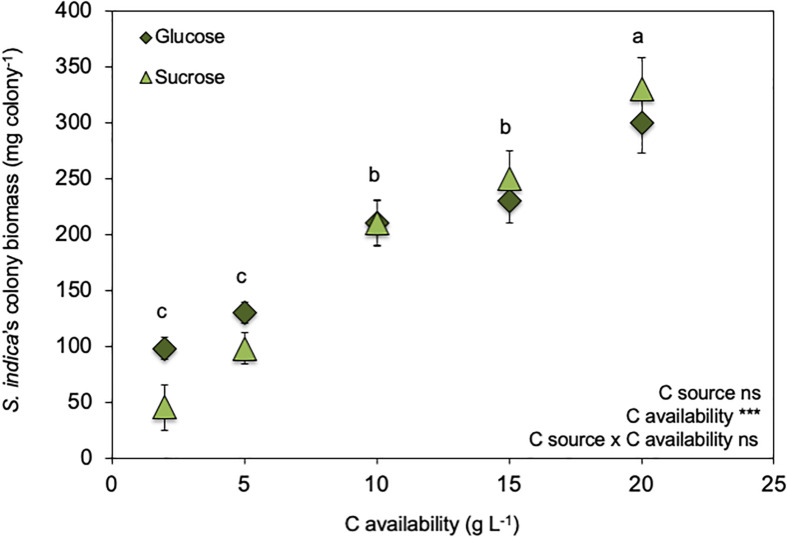
Effect of increasing C availabilities (glucose or sucrose) on *Serendipita indica*’s colony biomass (free-living stage). *** shows significant effects (*p* < 0.01) and “ns” shown non-significant (*p* > 0.05). Different letters show significant differences between C availabilities (*p* < 0.05). Symbols are the mean of 10 colonies per replicate (*n* = 3) ± SD.

Since we had observed that *S. indica*‘s characteristics during the free-living stage were similar when grown with both C sources (glucose and sucrose – Table 1 and Figures 1, 4), we tested the effect of inoculating wheat (*Triticum aestivum*) seedlings with *S. indica* grown only with the most widely used C source (glucose). We tested the same 5 increasing C availabilities (2, 5, 10, 15, and 20 g L^–1^) during *S. indica*’s free-living stage, plus a control treatment without *S. indica* inoculation. Each of the six treatments was replicated five times (6 treatments × 5 pots × = 30 pots).

**TABLE 1:** Effect of two contrasting C availabilities (2 and 20 g L^–1^ of glucose or sucrose) on *Serendipita indica*’s free-living stage phenotype.

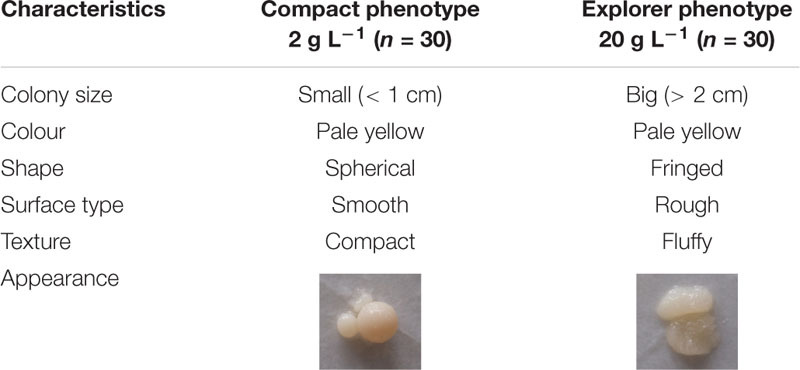

*The two C sources originated similar phenotypes. Characteristics were studied in 10 colonies per replicate (*n* = 3).*

#### Pot Experiment: Experimental Conditions, Plant Growth and Harvest

Wheat (*Triticum aestivum*) seeds, in a proportion of 100 mg of seeds to 100 mL of solution, were surface sterilised for 2 min in 70% ethanol solution (v/v) followed by 5 min in a NaClO solution (5%, v/v), and finally washed three times with sterile water.

Seeds were germinated on Petri dishes, at 15°C in the dark. After germination, five seedlings were transplanted to 1 L pots containing 600 mL of sterilised farming soil (pH 6.8; sandy loam texture; 2% organic matter) collected at Elvas Experimental Station for Plant Improvement (38° 53′ 44.07″ N, 7° 08′ 32.57″ W, Portugal) where they conduct efficacy field trials with wheat. The soil had been autoclaved (121°C for 1 hour, at 1 atm) twice on consecutive days and then left untouched for 1 week.

As previously described, *S. indica* cultures were grown in liquid modified Hill–Kaefer (KM) broth medium pH 6.5 (Hill and Kaefer, 2001), at 28°C and 125 rpm for 20 days, with three biological replicates per glucose availability. Mycelium was then separated from the culture medium, washed with sterile distilled water and dried using filter paper. Mycelia were homogenised in 40 mL of sterile NaCl solution 0.85% (w/v), and the homogenate analysed under the microscope to determine spore concentration.

Five days after transplant, seedlings were inoculated or not with *S. indica* by adding 1 mL of diluted inoculum next to the roots (approximately 15 spores per plant). Plants were grown in a walk-in growth chamber (model 5000EH, Aralab, Portugal) under controlled conditions (photoperiod 16 h/8 h, 25°C/20°C and 70% relative humidity) and the soil was maintained at 40–60% of soil pore space.

Plants were harvested 27 days after the inoculation. Roots were carefully washed with tap water and stored at 4°C for determining root colonisation or −20°C for determining potential enzymatic activities. Shoots were dried at 60°C until constant weight for biomass determination.

#### Wheat Roots Fungal Colonisation and Potential Extracellular Enzyme Activities

Inoculated wheat roots were analysed for their root *S. indica* colonisation. Root samples were cleaned by heating in 10% KOH solution for 60 min, then rinsed in water and stained by simmering in 0.02% trypan blue for 20 min. Then, the excess stain was removed in 50% lactophenol for 1–2 h prior to observation. Twenty root segments of 1 cm length were randomly chosen from each treatment and examined under a light microscope. Root colonisation was determined using the following formula: Root colonisation (%) = [(number of colonised segments/total number of segments examined) × 100] (Bertolazi et al., 2019).

Inoculated wheat roots were also analysed for their potential extra cellular enzyme activities related with the decomposing potential of C (β-glucosidase, β-glucuronidase, β-xylosidase and cellobiohydrolase), N (N-acetylglucosaminidase and Leucine aminopeptidase), and phosphorus (acid phosphatase) as previously described for *S. indica*‘s mycelia. Decomposing potentials of C and N corresponded to the sum of the respective enzyme activities. After the seventh enzyme assay, roots were washed with sterile distilled water, and dried at 50°C until constant weight. Enzyme activities were calculated based on the emitted fluorescence and were expressed as μmol h^–1^ of released substrate per gramme of root (Pritsch et al., 2011).

### Statistical Analysis

The effect of the C availability (glucose) on fungal potential extracellular enzyme activities and plant parameters (biomass and root fungal colonisation and potential extracellular enzyme activities) was tested separately using a one-way analysis of variance, with C availability as fixed factor. The effect of the C availability on fungal parameters (colony biomass, proportion of small spores, N concentration and isotopic signatures) was tested separately using a two-way analysis of variance, with C availability and C source as fixed factors. Bonferroni *post hoc* multiple comparisons tested for differences (*p* < 0.05) in fungal and plant parameters between C availabilities (including control plants for plant biomass). The effect of the C availability (glucose) on fungal H^+^ transport parameters was tested separately by Student *t*-test (*p* < 0.05). In all cases, preliminary analyses were performed to ensure that there was no violation of statistical assumptions (including the Levene’s test to check for homogeneity of variances). SPSS (version 23⋅0, IBM, Inc., Chicago, IL, United States) was used for all these analyses.

## Results

### Effect of the C Source and Availability During the Free-Living Stage on *S. indica*’s Phenotype

**FIGURE 2:**
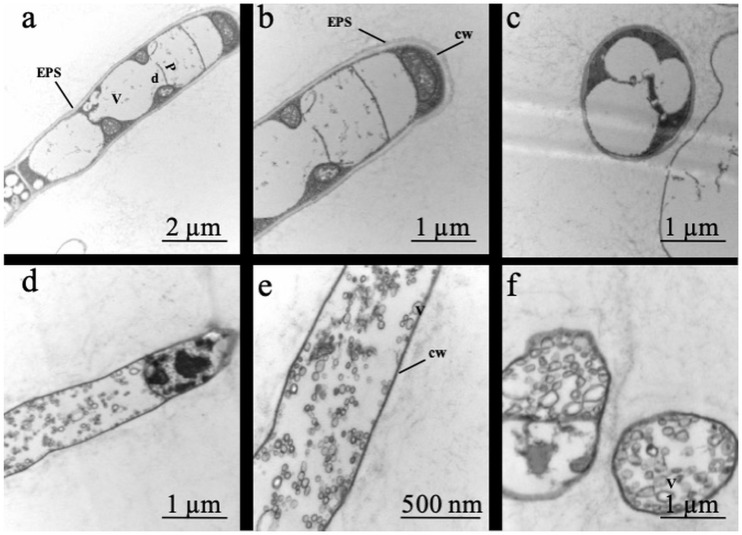
Effect of two contrasting C availabilities on *Serendipita indica*’s ultrastructure (free-living stage): 2 g L^–1^ of glucose **(a–c)** and 20 g L^–1^ of glucose **(d–f)**. V, vacuoles; d, dolipores; P, parenthosomes; cw, cell wall; ESP, extracellular polymeric substances.

**FIGURE 3:**
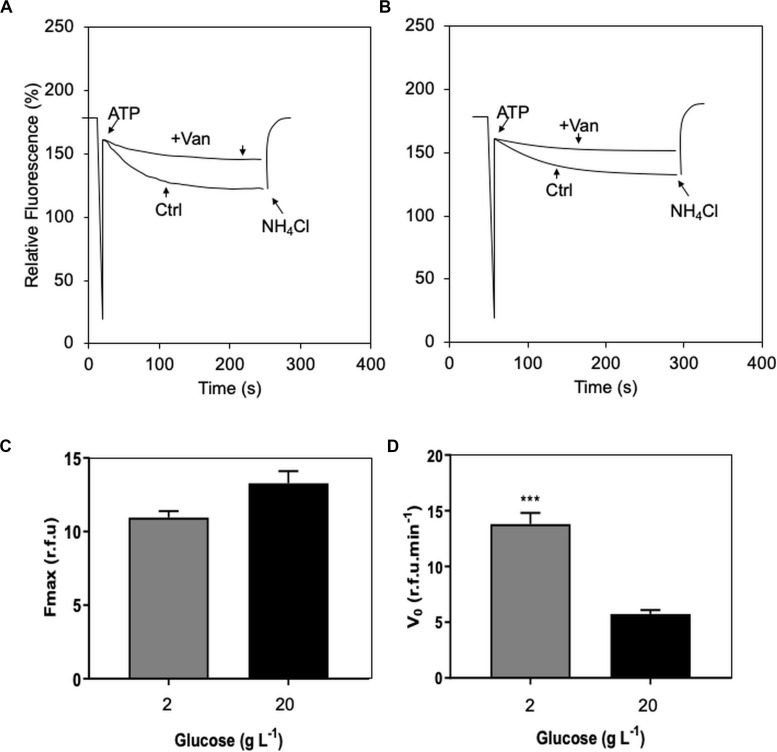
Effect of two contrasting C availabilities (2 and 20 g L^–1^ of glucose) on *Serendipita indica*’s (free-living stage) proton (H^+^) transport of plasma membrane ATPase (P-H^+^-ATPase): vanadate inhibition curve of the treatment of 2 g L^–1^ glucose **(A)**; vanadate inhibition curve of the treatment of 20 g L^–1^ **(B)**; maximum fluorescence (F_max_ – **C**), and initial velocity (V0) of P-H^+^-ATPase H^+^ transport **(D)**. CTR, control; + Van, preincubation with 0.2 M vanadate. *** shows significant difference by Student’s *t* test (*p* < 0.01). Bars are the mean of 10 colonies per replicate (*n* = 3) ± SD.

**FIGURE 4:**
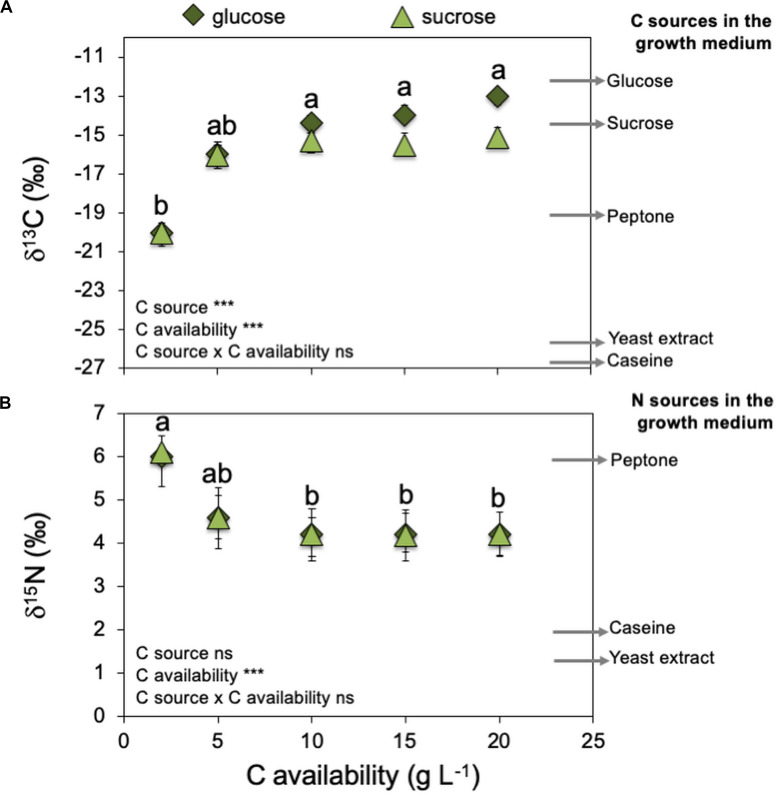
Effect of increasing C availabilities (glucose or sucrose) on *Serendipita indica*’s (free-living stage) stable isotopic signatures of C (δ^13^C – **A**) and N (δ^15^N – **B**). Arrows on the right side of the graphs indicate δ^13^C and δ^15^N of several medium components that can be C or N sources for *S. indica*. Arrows are the mean of three measurements per medium component (*n* = 3). *** shows significant effects (*p* < 0.01) and “ns” shown non-significant (*p* > 0.05). Different letters show significant differences between C availabilities (*p* < 0.05). Symbols are the mean of 10 colonies per replicate (*n* = 3) ± SD.

C availability in the growth medium (2–20 g L^–1^) regulated *S. indica*’s growth (Figure 1 and Supplementary Figure S1) ultrastructure (Figure 2) and physiology (Table 2, Figures 3, 4, and Supplementary Figure S2), resulting in two distinct phenotypes: compact and explorer (Table 1). This effect was independent of the C source (glucose or sucrose).

**TABLE 2:** Effect of increasing C availabilities (glucose or sucrose) on the potential enzymatic activities of *Serendipita indica*’s free-living stage.

**[C] (g L**^–^**^1^)**	**2**	**5**	**10**	**15**	**20**
**C cycling**					
β-gluco*	110.613.1a	48.75.9b	62.012.2b	42.55.9b	44.22.2b
β-xilo*	28.16.8a	12.31.4ab	8.41.4b	5.52.4b	5.02.2b
β-glucu*	3.72.3a	1.60.3ab	2.21.0ab	0.70.2b	0.70.1b
CeloBio*	36.82.3a	16.02.9b	16.71.5b	16.11.7b	15.82.8b
**N cycling**					
N-acetyl*	5.71.1b	8.53.1b	26.71.5a	21.53.5a	21.02.3a
Leu*	7.61.8b	7.82.1b	28.81.4a	25.81.0a	25.50.0a
**P cycling**					
AP*	27.012.9b	29.15.5b	43.111.3ab	57.22.7a	52.92.9a

Potential enzymatic activities were expressed as μmol h^–1^g^–1^ mycelium. * shows significant effects among enzyme activities (*p* < 0.01). Different letters show significant differences between C availabilities (*p* < 0.05). Values are the mean of 10 determination*s* per replicate (*n* = 3) ± SD. β-gluco, β-glucosidase; β-xilo, β-xylosidase; β-glucu, β-glucuronidase; CeloBio, celobiohydrolase; N-acetyl, N-acetylglucosaminidase; Leu, leucine aminopeptidase; and AP, acid phosphatase.

#### Growth and Ultrastructure

*Serendipita indica*’s colony biomass increased with increasing C availability (glucose or sucrose) in the growth medium (Figure 1). However, when C availability in the growth media was ≥ 10 g L^–1^, *S. indica* colonies became larger, their surface switched from smooth into rough, and their texture switched from ‘compact’ to ‘fluffy’ (data not shown). Further, when grown under C (glucose or sucrose) availability < 10 g L^–1^, *S. indica* cultures tended to present a smaller proportion of small spores (16–45 μm) when compared with cultures grown under higher C availability (Supplementary Figure S1). Again, this effect on spore size was independent of the C source (glucose or sucrose).

Analysis of the fungal ultrastructure showed that when grown under low C availability (2 g L^–1^ of glucose), *S. indica* had an extracellular layer of polymeric substance covering the hyphae, thick cell wall, large vacuoles occupying the cytoplasmic space and a clear distinction between the region of the parenthosome and that of the dolipore (Figure 2). By contrast, when grown under high C availability (20 g L^–1^ of glucose), *S. indica* displayed alterations in its conformation and internal organisation: no extracellular layer of polymeric substance, presence of a thin cell wall and of several cytoplasmic vesicles, and a difficult distinction of the dolipore region of glucose storage.

As a result of the above-mentioned characteristics, colonies which had been grown under low C availability (2 g L^–1^) were assigned to the compact phenotype while those which had been grown under high C availability (≥ 10 g L^–1^) were assigned to the explorer phenotype (Table 1). At 5 g L^–1^ we observed the co-existence of the two phenotypes (data not shown) and tested lower C availabilities (1, 2, 3, 4, 5, 6, 7, 8, 9, 10 g L^–1^) which showed that: (1) for C availabilities ≤ 4 g L^–1^, all *S. indica* colonies were compact; (2) at 5 g L^–1^, the two phenotypes co-existed; and (3) for C availabilities ≥ 6 g L^–1^, all *S. indica* colonies were explorer (data not shown).

#### Physiology

H^+^ transport analysis of membrane vesicles isolated from *S. indica* showed that preincubation with vanadate (a reversible inhibitor of these specific proton pumps – Wang et al., 2013) reduced the *F*_max_ by 70%, thus confirming significant plasma membrane ATPase (P-H^+^-ATPase) activity in this fungus (Figure 3). This reduction in F_max_ was independent of the C availability (2 and 20 g L^–1^ of glucose) under which the fungus had been grown (Figure 3C). However, the initial transport velocity of H^+^ (V_0_) was 2.4 times higher in the compact phenotype (grown under 2 g L^–1^ of glucose) than in the explorer (grown under 20 g L^–1^ of glucose), suggesting that excess of glucose may negatively influence the efficiency of P-H^+^-ATPase activity in *S. indica* (Figure 3D).

Increasing C (glucose or sucrose) availability in the growth medium ≥ 10 g L^–1^ decreased *S. indica*’s mycelium nitrogen (N) concentration (Supplementary Figure S2). Therefore, the compact phenotype presented higher N concentrations (6.8 and 6.0% N) than the explorer phenotype (4.9 and 5.3% N). When the fungus was grown at 2 g L^–1^, there was no effect of the C source on *S. indica* colonies δ^13^C (Figure 4A), and the mycelium δ^13^C was compatible with the use of peptone (δ^13^C = −19.1) as the major C source, which could be confirmed by the mycelium δ^15^N also very similar to that of peptone (δ^15^N = 6.1 – Figure 4B). At 5 g L^–1^ of glucose or sucrose, the mycelium δ^13^C became more similar to that of glucose and sucrose, and the mycelium δ^15^N became less positive, possibly due to the use of other N sources (casein and yeast extract). At higher availabilities of glucose or sucrose (> 10 g L^–1^), mycelium δ^13^C was compatible with the main use of these sugars as C sources, and no changes were detected on mycelium δ^15^N, indicating that most probably *S. indica* was using the available N sources in the same proportion. These results show that the compact phenotype used more peptone as C and N sources, while the explorer phenotype used more glucose or sucrose as C source and casein and yeast extract as N source.

Low C availability (2 g L^–1^ of glucose) stimulated the potential activities of all the extracellular enzymes related with C cycling, while higher C availabilities (≥ 10 g L^–1^ of glucose) stimulated the potential activities of the extracellular enzymes related with N and P cycling (Table 2). Similar results were obtained for sucrose (data not shown). The compact phenotype has a higher decomposing potential for C while the explorer phenotype has a higher decomposing potential for N and P related substrates.

### Effect of *S. indica*’s Free-Living Stage Growth Conditions on Its Potential as PGPM During the Symbiosis Stage

**FIGURE 5:**
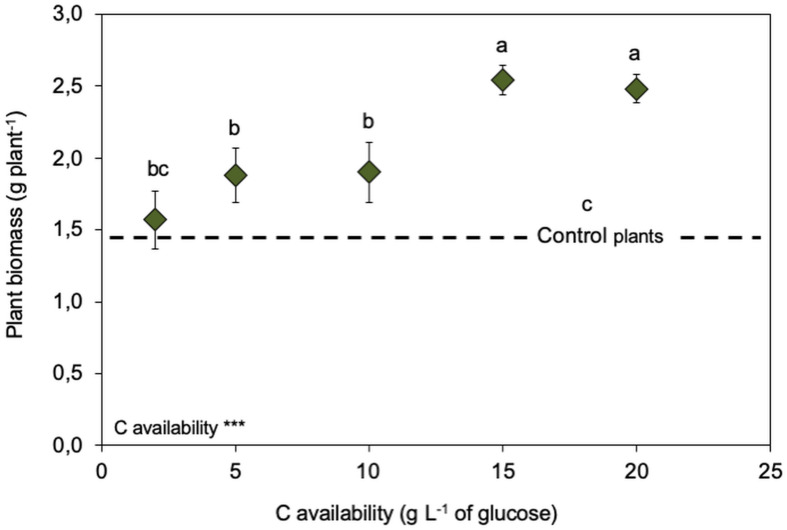
Legacy of increasing C availabilities (glucose) during the fungal free-living stage on biomass of *Serendipita indica*-inoculated wheat plants (symbiosis stage). Dashed line represents the biomass produced by non-inoculated plants. *** shows significant effects (*p* < 0.01) and “ns” shown non-significant (*p* > 0.05). Different letters show significant differences between C availabilities (*p* < 0.05). Symbols are the mean of 10 colonies per replicate (*n* = 3) ± SD.

Inoculating the endophytic fungus *S. indica* promoted the growth of inoculated wheat plants compared to non-inoculated ones (control plants – Figure 5). However, there was an effect of the free-living stage growth conditions (C availability), as plant growth promotion by *S. indica* was stronger (almost twice the biomass of control plants) when *S. indica* had been grown under high C availability (15 and 20 g L^–1^ of glucose – explorer phenotype). *S. indica* root colonisation was similar for all treatments and was around 70–75% (data not shown). Quantifying the potential activities of extracellular enzymes of the inoculated roots showed a decrease in β-glucuronidase (involved in C cycling) when the fungus had been grown under high C availability (15 and 20 g L^–1^ of glucose – explorer phenotype – Table 3). By contrast, the root extracellular enzymes involved in the N (N-acetyl-glucosaminidase and leucine aminopeptidase) and P (acid phosphatase) cycling showed increased potential activities by one order of magnitude when the fungus had been grown under high C availability (≥ 10 g L^–1^ of glucose – explorer phenotype).

**TABLE 3:** Legacy of increasing C availabilities (glucose) during the fungal free-living stage on the potential enzymatic activities of the inoculated plant roots (symbiotic stage).

**[C] (g L**^–^**^1^)**	**2**	**5**	**10**	**15**	**20**
**C cycling**					
β-gluco	24.83.1	25.64.4	31.13.2	26.02.9	24.02.7
β-xilo	8.16.8	7.30.4	7.40.4	6.11.4	5.31.1
β-glucu*	1.80.3a	1.60.3a	1.30.3a	0.30.1b	0.40.1b
CeloBio	6.40.6	6.50.9	6.20.8	6.30.9	6.30.9
**N cycling**					
N-acetyl*	5.81.2b	6.43.0b	36.53.1a	41.03.6a	40.02.1a
Leu*	3.61.0b	3.82.2b	44.81.6a	45.01.1a	41.22.3a
**P cycling**					
AP	6.72.0b	10.11.5b	48.15.3a	61.06.7a	53.16.1a

Potential enzymatic activities were expressed as μmol h^–1^g^–1^ root. * shows significant effects among enzyme activities (*p* < 0.01). Different letters show significant differences between C availabilities (*p* < 0.05). Values Results are the mean of 10 determination per replicate (*n* = 3) ± SD. β-gluco, β-glucosidase; β-xilo, β-xylosidase; β-glucu, β-glucuronidase; CeloBio, celobiohydrolase; N-acetyl, N-acetylglucosaminidase; Leu, leucine aminopeptidase; and AP, acid phosphatase.

## Discussion

**FIGURE 6:**
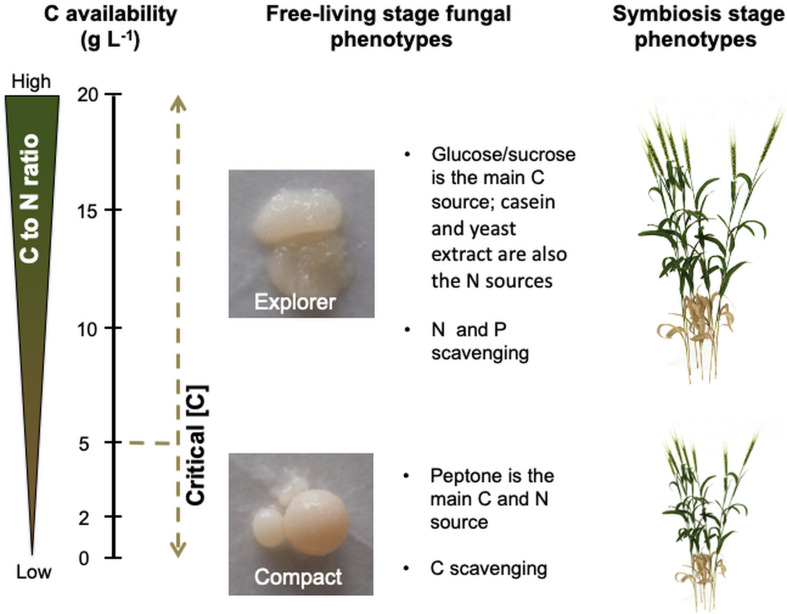
C availability in the growth medium triggers nutrient imbalances which regulate *Serendipita indica*’s phenotype during the free-living and symbiosis stages (when later colonising the plant host). Low C availability (below 5 g L^–1^ of glucose or sucrose) leads to C deprivation which triggers the C scavenging mode displayed by the compact phenotype. High C availability (above 5 g L^–1^ of glucose or sucrose) leads to N and P limitation which triggers the N and P scavenging mode displayed by the explorer phenotype. When *S. indica* colonises the plant host (symbiosis stage), the explorer phenotype provides greater benefits to plant growth than the compact phenotype.

By growing the endophytic fungus *S. indica* under increasing C availabilities we showed that there is a legacy of the conditions under which the fungus was grown, regulating its phenotype both in its free-living and symbiosis stages (Figure 6). This can be explained by the proportion of C to N in the growth medium, which regulates *S. indica*’s morphology and physiology originating two distinct phenotypes (compact and explorer), independently of the C source (glucose or sucrose).

### C Availability Regulates the Free-Living Stage Fungal Phenotype

*Serendipita indica*‘s ability to produce colonies with distinct phenotypes was already known (Kaldorf et al., 2005; Suman et al., 2010) but our results clearly show, for the first time, that C availability is one of the factors regulating colony phenotype: the compact phenotype seems characteristic of low C availability (< 5 g L^–1^) while the explorer, characteristic of high C availability (> 5 g L^–1^ – Tables 1, 2 and Figures 1–4). Each phenotype must provide the most suitable morphology to cope with the experienced environmental conditions. At higher C availability (glucose or sucrose), fungal growth is mostly limited by the availability of nutrients other than C (mainly N). So, it is possible that the explorer phenotype will be regulated to scavenge other essential nutrients increasing the contact area between the mycelium and the culture medium. Furthermore, C depletion (≤ 20 g L^–1^ of glucose) is known to stimulate *S. indica*’s sporulation, with chlamydospores starting to form with glucose exhaustion after 3–5 days of culture and continuing for another 3 days (Suman et al., 2010). This means that in our study, the sporulation process started earlier when *S. indica* was grown under lower C availability, and therefore at the same time point, spores formed under low C availability are older and more mature, bigger, than those formed at higher C availability which is supported by our observation of lower proportion of small spores under low C availability (Supplementary Figure S1).

Although glucose has been described as the preferential C source for *S. indica* growth (for concentrations < 56 mM or < ∼10 g L^–1^ – Kumar et al., 2011) in the range between 2 and 20 g L^–1^ colony biomass was similar in both glucose and sucrose supplemented growth media (Figure 1). *S. indica*‘s ability to use both glucose and sucrose is supported by genomic and transcriptomic analyses showing that it produces intra- and extracellular invertases and expresses sucrose membrane transport proteins (Zuccaro et al., 2011; Rani et al., 2016).

The study of morphological responses of *Wallemia* sp. fungi to high concentrations of sugar and honey showed that although they are xerophilic, when submitted to the osmolytes, these fungi presented ultrastructural alterations due to reduction in the extracellular polymer layer and formation of various cytoplasmic vesicles (Kralj Kuncic et al., 2013). In our study, growing *S. indica* under 20 g L^–1^ glucose also modified its ultrastructure (Figure 2) while for the xerophilic fungi *W. muriae* and *W. sebi*, adapted to adverse conditions, not even 20 g L^–1^ glucose resulted in major changes in their ultrastructure (Kralj Kuncic et al., 2013).

### C Availability Regulates Fungal Physiology in Its Free-Living Stage

Glucose availability is known to play an important role in the modulation and expression of P-H^+^-ATPase in yeast (Eraso and Gancedo, 1987; Portillo, 2000) and in filamentous fungi, including basidiomycetes (Ramos et al., 2005; Kralj Kuncic et al., 2013). Regarding fungi capable of associating with plants, although there is little information on the effect of glucose availability on P-H^+^-ATPase, both AMF- (Liu et al., 2016; Lopez-Coria et al., 2016; Aloui et al., 2018) and *S. indica*-colonised plants (Bertolazi et al., 2019) have shown increased nutrient uptake due to the increase in activity and expression of these proton pumps.

In yeast, adding glucose rapidly increases the transcription of the gene encoding the P-H^+^-ATPase (PMA1 gene), and its product (Rao et al., 1993; Castanheira et al., 2018). Further, when yeast had been grown without glucose, adding this carbohydrate rapidly increases P-H^+^-ATPase activity due to increased Vmax, an effect that is fully reversible when glucose is removed (Serrano, 1983). The 2.4-fold reduction in the initial rate (*V*_0_) of P-H^+^-ATPase we observed suggests that high glucose availability (20 g L^–1^) may decrease the efficiency of *S. indica*‘s P-H^+^-ATPase activity (Figure 3D). This means that at least high glucose availability does not positively regulate P-H^+^-ATPase activity in *S. indica*, as shown for *Penicillium simplicissimum* (Burgstaller et al., 1997), the filamentous fungi *Aspergillus nidulans* and *Neurospora crassa* (Abdallah et al., 2000). On the other hand, high glucose availability (20 g L^–1^) resulted in a 14% increase in F_max_, suggesting increased P-H^+^-ATPase activity (Figure 3C). Although further analysis confirming our data and testing more glucose availabilities are required, our study is the first to consider bioenergetics modulation by one of the most assimilated energy sources by *S. indica* in the symbiotic process with wheat plants.

The nutritional composition of *S. indica*’s mycelia seems to reflect the nutrient imbalances and limitations which resulted from increasing C availability: when C is limiting, the mycelium is enriched in N, while at higher C availabilities, N concentration decreases to values that may limit fungal growth (Supplementary Figure S2). These results show that both phenotypes differ in their nutritional composition (at least for N), with the compact phenotype being enriched in N relatively to the explorer phenotype. Part of the N enrichment of the compact phenotype may be explained by the use of alternative C sources that also contain N, namely peptone, casein, and yeast extract components. This hypothesis was corroborated by *S. indica*’s natural isotopic signatures (δ ^13^C and δ ^15^N – Figure 4). When grown under low C availability (2 g L^–1^) the mycelium δ^13^C and δ^15^N were very similar to those of the peptone used in the growth media, supporting the use of peptone as C and N sources under low C availability. Based on the mycelium δ^13^C and δ^15^N is seems that when available, *S. indica* uses glucose or sucrose as preferential C sources, and under these circumstances a higher proportion of N is coming from casein and yeast extract components. Since major differences between compact and explorer phenotypes involve N sources (Figure 4) and concentration (Supplementary Figure S2) it is possible that the PiAMT1 gene expression is involved in this regulation, similarly to what happens in yeast. In *S. cerevisiae* the high affinity ammonium transporter MEP2 is involved in the transition from buddy to pseudohyphal growth in response to N starvation, via cAMP/PKA and MAPK cascade pathways (van den Berg et al., 2016). This interpretation is supported by *S. indica*’s extracellular potential enzyme activities which show that the catabolism of C polymers was higher at low C availability, while at higher C availability it was the N and P scavenging enzyme activities that were higher (Table 2).

### Legacy of *S. indica*’s Free-Living Stage Growth Conditions on Its Potential as PGPM During the Symbiosis Stage

Considering the effects of inoculating wheat plants with *S. indica* grown *in vitro* under increasing C availabilities, it is clear that this endophytic fungus promoted plant growth, with inoculated plants growing more than non-inoculated ones (Figure 5). But the effect on plant growth promotion was almost double when the fungus had been grown under high C availability (15 and 20 g L^–1^ of glucose, displaying the explorer phenotype). This different plant growth promotion could not be attributed to differences in *S. indica*’s root colonisation, as root colonisation was similar for all treatments (data not shown).

Given that *S. indica* is known for promoting plant growth (Varma et al., 1999; Singh et al., 2000; Qiang et al., 2012; Varma et al., 2012; Li et al., 2017; Xu et al., 2018) mainly by improving plant N and P uptake (Achatz et al., 2010; Aslam et al., 2019), the highest plant growth promotion when wheat plants were inoculated with *S. indica* explorer phenotype may be related with its higher potential for N and P scavenging. In fact, the potential activity of the extracellular enzymes of the colonised roots showed a reduction of the decomposing potential of C, accompanied by an increment of the decomposing potential for N and P substrates (Table 3). These observations are in line with the putative mechanisms involved in the symbiotic partnership where *S. indica* may receive C from the plant in exchange for the supply of N and/or P (Varma et al., 2012). What is interesting, and deserves further investigation, is the legacy of *S. indica*’s free-living stage growth conditions on its potential to promote plant growth (Figure 5) and improve N and P potential scavenging (Table 3) during the symbiosis stage. This is especially relevant because when colonising some plant species, *S. indica* displays different behaviours in different symbiotic stages; it can switch from biotroph to saprotroph in later stages of colonisation (Lahrmann et al., 2013). *S. indica*’s saprophytic behaviour on later stages of barley roots’ colonisation is associated with the induction of the fungal genes encoding for hydrolytic enzymes and C and N transporters, namely the high affinity ammonium transporter PiAMT1 which functions as N sensor under N deficiency conditions. Further, Kaldorf et al. (2005) observed that when *S. indica* had been grown during its free-living stage in the presence of ammonium, the interaction between *S. indica* and *Populus* Esch5 changed from mutualism (when grown without ammonium) towards antagonism (when grown in Woody Plant medium with ammonium). The authors postulated that this change from mutualism towards antagonism was related with the existence of one or more toxic compounds synthesised by *S. indica* when grown in the presence of ammonium. In our study we did not observe any symptom of toxicity (or other negative effects such as nutrient deficiency) when wheat plants were inoculated with the two *S. indica*’s phenotypes. We cannot exclude the possibility that the lower plant growth promotion by the compact phenotype could be related with the accumulation of toxins as postulated by Kaldorf et al. (2005), but it does not seem very likely as the growth medium we used (modified KM medium – Hill and Kaefer, 2001) does not contain ammonium. Also, as far as we are aware, no toxic compound produced by *S. indica* when grown in the presence of ammonium was ever identified. Instead, it seems that by activating the high affinity ammonium transporter PiAMT1 in *S. indica*, ammonium can trigger the activation of *S. indica*’s saprophytic programme during the symbiosis stage (Lahrmann et al., 2013).

### Implications for Plant-Growth- Promoting-Microbes’ Production

Plant growth-promoting microbes, formulated as inoculant biofertilizers, show strong potential by improving nutrient use efficiency. However, the practical use of PGPM/biofertilizers by farmers remains limited because of inconsistent results (Rose et al., 2014). Many factors can contribute to this inconsistency (e.g., being isolated from a given crop, PGPM are not equally efficient/beneficial to all other crops, and conditions (abiotic and biotic) can affect the plant-PGPM symbiosis; Ji et al., 2019). But evidence is starting to appear showing that developing a consistent PGPM/biofertilizer is not just isolating the microbe with the desired traits and propagate it massively. For instance, the legacy of the farming system (conventional or organic) has been shown to have a significant effect on the physiology of phosphate solubilising bacteria isolated from the rhizospheres of *Carica papaya* (Melo et al., 2016), and on the interactions among these bacteria (Melo et al., 2018). Further, this study with phosphate solubilising bacteria showed that the output of the interactions among microbes (cooperation or antagonism) was not phylogenetically determined (Melo et al., 2018). Another example showed that as the soil microbial community interfered with AMF’s benefits for plant growth and these benefits depended on the AMF species, the soil microbial community and AMF species should be taken into consideration when applying AMF inoculants in agriculture (Dias et al., 2018). Here, we showed that there is a legacy of the conditions under which *S. indica* was grown, regulating its phenotype both in the free-living and symbiosis stages (Figure 6). The importance of the legacy of the conditions under which *S. indica* was grown had previously been shown for growth media containing or not ammonium (Kaldorf et al., 2005), Although these examples need to be confirmed under field trials, they highlight the importance of considering microbial ecology in designing PGPM/biofertilizer.

## Conclusion

Besides the macromorphological differences, the two phenotypes also differed physiologically: the compact phenotype displayed a higher decomposing potential (saprophytic lifestyle) than the explorer phenotype (biotrophic lifestyle), so that glucose depletion in the culture medium was a stimulus for the secretion of organic matter hydrolysing enzymes. Therefore, our results suggest that *S. indica* phenotypic and physiological plasticity is modulated by the C to N proportion and depending on its life stage, the fungus may present different nutritional demands: when on a free-living stage, C depletion would be a stronger signal than N starvation for the fungus to increase the secretion of C related hydrolytic enzymes, while during symbioses, when C is supplied by the plant, N starvation would be the major stimulus for *S. indica* saprophytic behaviour as described by Lahrmann et al. (2013). However, these different nutritional demands depending on *S. indica*’s life stage remain to be confirmed.

Also, further studies are needed to test the different *S. indica* phenotypes under more extreme conditions, where the hosts will really benefit from each phenotype’s potential, and to understand if the characteristics acquired *in vitro* persist under field conditions. The lack of these studies, especially the lack of field trials, limits our study’s contribution to the development of efficient *S. indica*-based PGPM, either *S. indica* alone or in consortium with bacteria. Indeed, studies demonstrating interactions between *S. indica* and soil bacteria are accumulating in recent years: (i) *S. indica*-bacteria interactions can benefit (Jiang et al., 2018; Alabid et al., 2019) or affect (Leyva-Rojas et al., 2020) *S. indica*’s growth during the free-living stage; and (ii) *S. indica*-bacteria interactions have been shown to enhance crop production (Guo et al., 2017; del Barrio-Duque et al., 2019; Dabral et al., 2020) and resistance to phytopathogens (del Barrio-Duque et al., 2019, 2020) and salt stress (Heidarianpour et al., 2020). This recent trend further reinforces the need for synergy between fungal and bacterial endophyte research efforts (Le Cocq et al., 2017), especially taking into consideration the bacteria intimately associated with *S. indica* (Sharma et al., 2008; Guo et al., 2017; del Barrio-Duque et al., 2020).

## Data Availability Statement

All datasets generated in this study are included in the article/Supplementary Material.

## Author Contributions

TD, AV, ACR, and CC conceived and designed the experiments and prepared the manuscript. RC, AAB, JM, and MC conducted the experiments (both free-living stage and symbiosis stage) and characterised the fungal phenotypes. FE and FLO conducted the microscopy studies. VP, ACR, and SBS extracted, isolated, and determined the plasma membrane proton pumps activities. RC, AAB, CM, and ACR measured potential enzymatic activities. TD, SBS, FE, and ACR analysed the data. All authors contributed to the discussion and approved the final manuscript.

## Conflict of Interest

The authors declare that the research was conducted in the absence of any commercial or financial relationships that could be construed as a potential conflict of interest.
