# Supplementary material for: The Free-Living Stage Growth Conditions of the Endophytic Fungus Serendipita indica May Regulate Its Potential as Plant Growth Promoting Microbe
Source: Front Microbiol. 2020 Sep 22;11:562238. doi: 10.3389/fmicb.2020.562238 (PMC7536269; doi:10.3389/fmicb.2020.562238)
Supplement: Supplementary file 1 [file Table_1.DOCX]

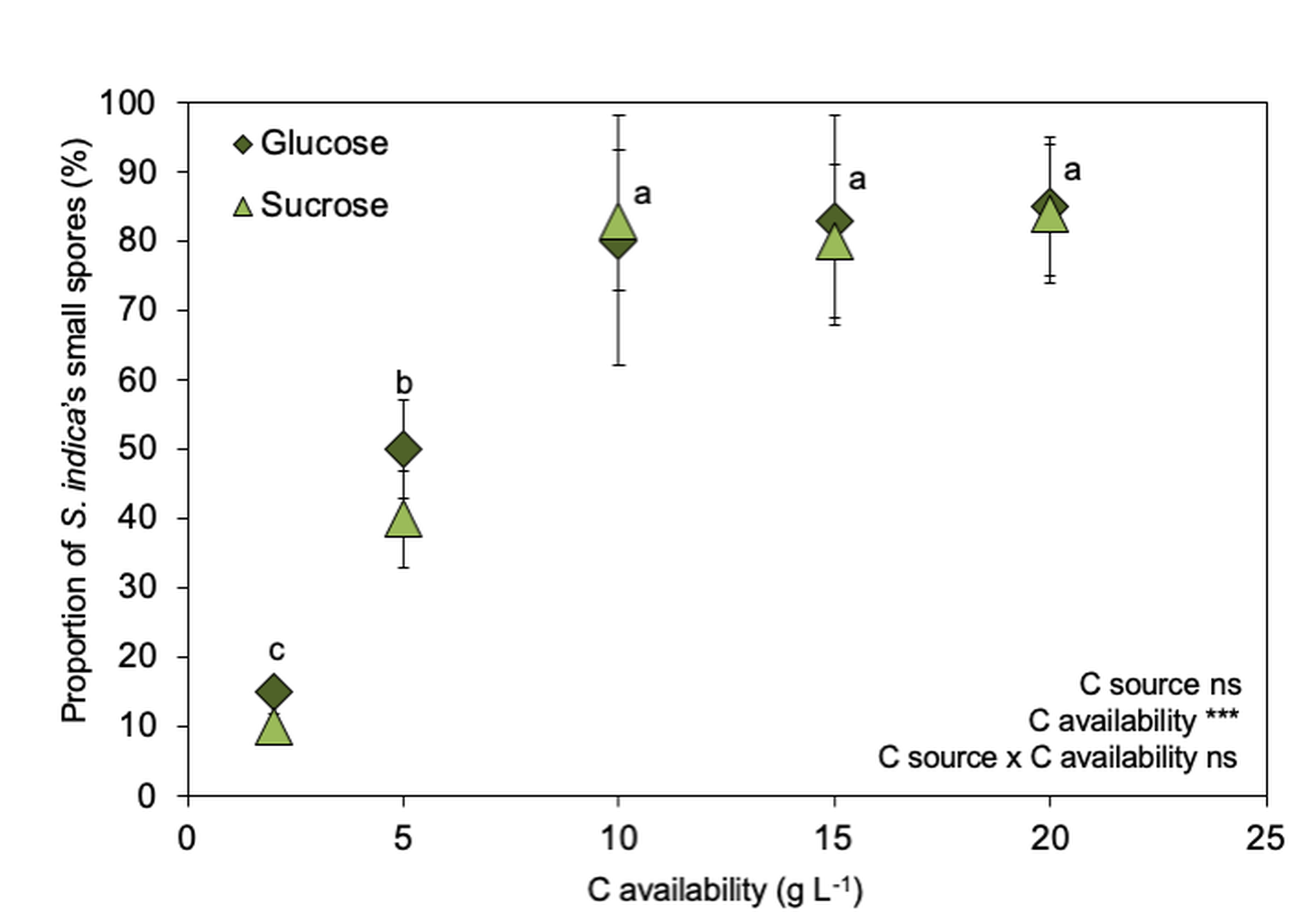


**Figure S1** – Effect of increasing C availabilities (glucose or sucrose) on the proportion of *S. indica*’s small to large spores. *** shows significant effects (*p* < 0.01) and “ns” shown non-significant (*p* > 0.05). Different letters show significant differences between C availabilities (*p* < 0.05). Symbols are the mean of 10 colonies per replicate (n=3) ± SD.


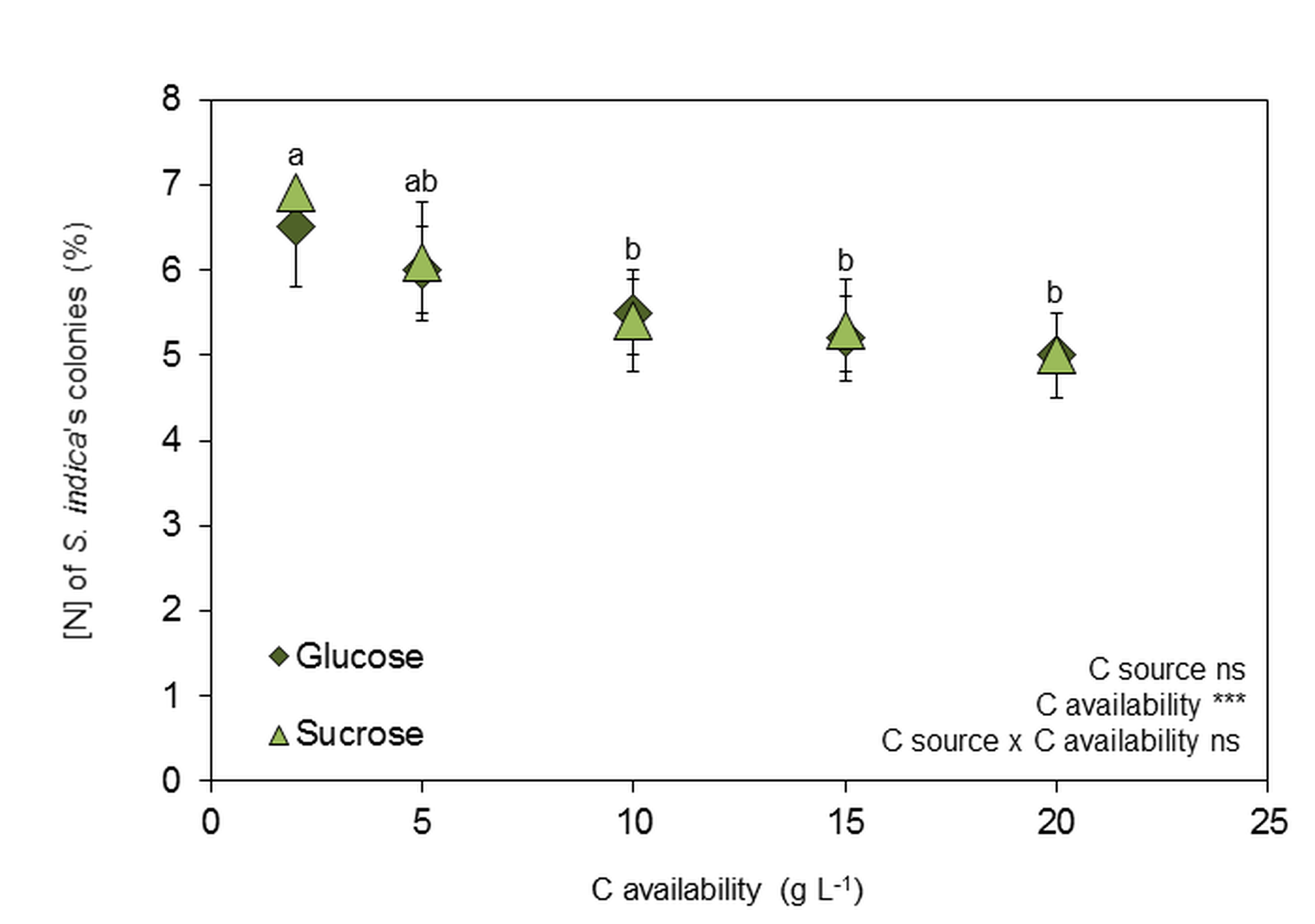


**Figure S2** – Effect of increasing C availabilities (glucose or sucrose) on the N concentration of *S. indica*’s colonies. *** shows significant effects (*p* < 0.01) and “ns” shown non-significant (*p* > 0.05). Different letters show significant differences between C availabilities (*p* < 0.05). Symbols are the mean of 10 colonies per replicate (n=3) ± SD.
